# Hepatic Alterations in a BTBR T + Itpr3tf/J Mouse Model of Autism and Improvement Using Melatonin via Mitigation Oxidative Stress, Inflammation and Ferroptosis

**DOI:** 10.3390/ijms25021086

**Published:** 2024-01-16

**Authors:** Rita Rezzani, Marzia Gianò, Daniela Pinto, Fabio Rinaldi, Cornelis J. F. van Noorden, Gaia Favero

**Affiliations:** 1 Anatomy and Physiopathology Division, Department of Clinical and Experimental Sciences, University of Brescia, 25123 Brescia, Italy; marzia.giano@unibs.it (M.G.); gaia.favero@unibs.it (G.F.); 2 Interdipartimental University Center of Research “Adaption and Regeneration of Tissues and Organs (ARTO)”, University of Brescia, 25123 Brescia, Italy; 3 Italian Society for the Study of Orofacial Pain (Società Italiana Studio Dolore Orofacciale-SISDO), 25123 Brescia, Italy; 4 Human Microbiome Advanced Project Institute, 20129 Milan, Italy; dpinto@giulianipharma.com (D.P.); fabio.rinaldi@studiorinaldi.com (F.R.); 5 Department of Genetic Toxicology and Cancer Biology, National Institute of Biology, 1000 Ljubljana, Slovenia; c.j.vannoorden@nib.si

**Keywords:** autism spectrum disorder, liver, oxidative stress, inflammation, ferroptosis

## Abstract

Autism spectrum disorder (ASD) is a complicated neurodevelopmental disorder, and its etiology is not well understood. It is known that genetic and nongenetic factors determine alterations in several organs, such as the liver, in individuals with this disorder. The aims of the present study were to analyze morphological and biological alterations in the liver of an autistic mouse model, BTBR T + Itpr3tf/J (BTBR) mice, and to identify therapeutic strategies for alleviating hepatic impairments using melatonin administration. We studied hepatic cytoarchitecture, oxidative stress, inflammation and ferroptosis in BTBR mice and used C57BL6/J mice as healthy control subjects. The mice were divided into four groups and then treated and not treated with melatonin, respectively. BTBR mice showed (a) a retarded development of livers and (b) iron accumulation and elevated oxidative stress and inflammation. We demonstrated that the expression of ferroptosis markers, the transcription factor nuclear factor erythroid-related factor 2 (NFR2), was upregulated, and the Kelch-like ECH-associated protein 1 (KEAP1) was downregulated in BTBR mice. Then, we evaluated the effects of melatonin on the hepatic alterations of BTBR mice; melatonin has a positive effect on liver cytoarchitecture and metabolic functions.

## 1. Introduction

Autism spectrum disorder (ASD) is a heterogeneous, disabling neurodevelopmental disorder that affects social interactions, limits interests and induces stereotype behavior [1,2,3]. The prevalence of ASD is increasing and creates a social burden. Currently, psychostimulant and antipsychotic interventions are the only therapeutics available to treat ASD patients [4].

The etiology of ASD is not well understood [2,5], but it is likely that both biological and environmental factors are involved [6]. Biologically, genetic and nongenetic factors play a role, such as immunological and mitochondrial malfunction associated with oxidative stress, neurotrophic dysregulation and inflammation [7,8,9,10]. There is increasing evidence that ASD patients show excessive reactive oxygen species (ROS) production [8,11,12]. Environmental factors involve, among others, exposure to both essential and toxic metals [13,14,15]. Dysregulated iron metabolism, causing an increased availability of free iron, can significantly impact hepatic metabolism [16]. Free iron has been implicated in inducing amyloid-β plaques, contributing to toxicity via oxidative stress in Alzheimer’s disease and other neurodegenerative diseases [17].

An autistic mouse model, BTBR T + Itpr3tf/J (BTBR) mice, originally developed to study insulin resistance, diabetes-induced nephropathy and phenylketonuria, has been identified to consistently display autism-like behavior [18]. This animal model is considered a translational tool to evaluate potential therapies for ASD [18,19]. Recently, liver disorders have become a significant health concern and are an issue commonly found in autistic children [9,20]. Moreover, Trinchese et al. [8] showed that inflammation and oxidative stress in these mice induce mitochondrial dysfunction and mitochondrial fission in the liver. Hepatocytes are rich in mitochondria that produce ROS [21], and exposure to ROS induces oxidative stress, which results in lipid peroxidation and reduced antioxidant activity, causing liver damage [22,23] and, subsequently, leading to liver diseases [24,25].

Thus far, the role of the liver in ASD has been poorly investigated. Therefore, we studied the crosstalk between alterations in hepatic functions and ASD. Liver morphology was analyzed with haematoxylin-eosin staining and a common and reliable dye for detecting iron in tissues (Perls staining), and liver functions were analyzed by staining the biological markers of oxidative stress and inflammation, and the master regulators of a recently discovered form of cell death called *ferroptosis* in BTBR mice [16,18,26,27]. Ferroptosis is a recently discovered iron-dependent type of cell death characterized by ROS accumulations in cells, leading to Fe ion deposition and lipid peroxidation that results in cell death [18]. It is a result of redox inequity between the free iron-induced production of lipid hydroperoxides and various antioxidant defense systems, such as glutathione peroxidase 4 (GPX4), which detoxify free radicals and lipid oxidation products [18,28,29]. Moreover, ferroptosis has been implicated in multiple pathologies and is associated with neurodegeneration, nonalcoholic fatty liver diseases and cardiovascular diseases such as atherosclerosis [16]. Disruption of iron metabolism, in relation to the expression of the transcription factor nuclear factor erythroid-related factor 2 (NRF2), may significantly impact the ferroptosis pathway. It has been demonstrated that NRF2 is able to reduce anaerobic glycolysis and ROS levels in glioblastoma stem cells and leukemic stem cells [30]. Under physiological conditions, NRF2 resides in the cytoplasm, where it is attached to Kelch-like ECH-associated protein 1 (KEAP1), which negatively regulates NRF2 and keeps it in the cytoplasm [31]. Under oxidative stress conditions, NRF2 protein is stabilized and initiates a multistep pathway of activation that includes nuclear translocation [32]. After its translocation to the nucleus, NRF2 binds to antioxidant response elements and activates the transcription of cytoplasmic genes. Heme oxygenase-1 (HO-1) and other proteins are strictly under the control of NFR2 [33]. This pathway is considered to have a potential protective role of NFR2 in several types of cells, such as renal proximal tubular epithelial cells, leading to an upregulation of antioxidant enzymes such as superoxide dismutase 3 (SOD3) [33].

On the basis of these considerations, we report that ferroptosis participates in a spectrum of liver diseases, both acute and chronic [19,33], and we hypothesize that ferroptosis is also involved in hepatocyte alterations of BTBR mice. Only after considering the involvement of ferroptosis in the liver of BTBR mice, we evaluated the possible effect of an antioxidant, such as melatonin, in hepatic ASD. 

Moreover, BTBR mice were treated with melatonin, a potent antioxidant that has been shown to have many cellular protective effects in neurological and non-neurological disorders [34,35]. Melatonin (N-acetyl-5-methoxytryptamine) is an indoleamine synthesized from the amino acid tryptophan in the pineal gland and other organs, such as the liver [21,36,37,38]. Studies have extensively demonstrated the effects of melatonin on oxidative stress, lipid peroxidation, and its therapeutic potential in liver injuries and diseases, showing its beneficial effects [35]. A recent study demonstrated that melatonin attenuates hepatocyte ferroptosis induced by lead or lipopolysaccharide exposure through the activation of AMP-activated protein kinase phosphorylation [39]. However, it is crucial to acknowledge that the exploration of the relationship between melatonin and ferroptosis is in a nascent stage, and many uncharted fields require further investigation.

Therefore, the aims of the present study were (a) to analyze morphological and biological alterations in the liver of BTBR mice in combination with markers related to oxidative stress and inflammation and (b) to identify the potential association between ferroptosis-related mechanisms and morphological changes in the liver in the BTBR mice; (c) to evaluate and identify therapeutic strategies for targeting the liver and alleviating metabolic impairments using melatonin administration.

Our research hypothesis is that oxidative stress, inflammation and even ferroptosis may be important tools for ASD evaluation.

## 2. Results

### 2.1. Hepatic Histological Features

The BTBR and CTR experimental groups treated with the vehicle were respectively redefined as “BTBR mice” and “CTR mice” for the following morphological, morphometrical and immunohistochemical analyses.

**Figure 1:**
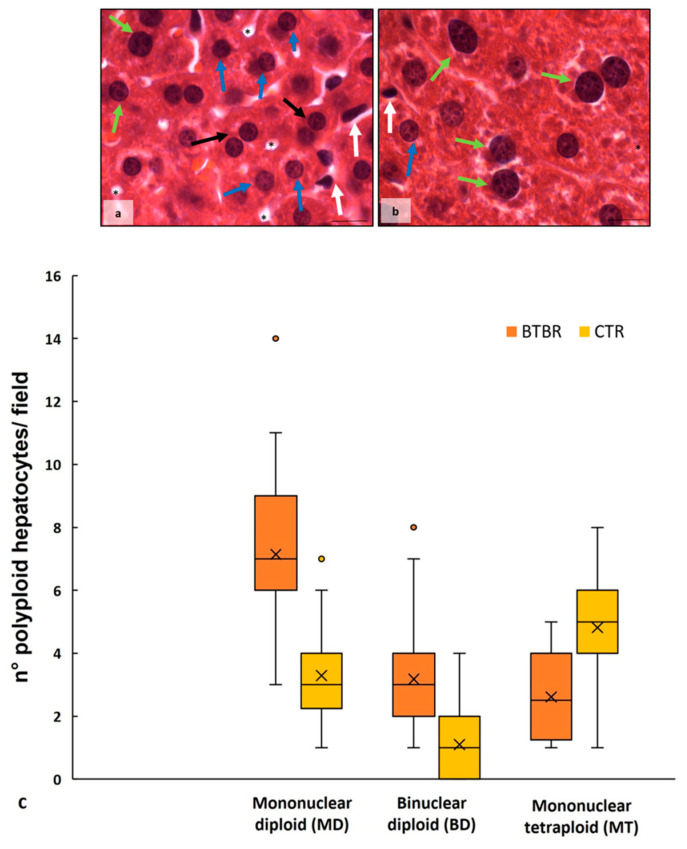
Hepatic histological evaluation. Representative photomicrographs of BTBR (**a**) and CTR (**b**) liver stained with haematoxylin-eosin. Bar: 10 µm. The black and white arrows show binuclear hepatocytes and Kupffer cells, respectively; the asterisks show the vacuoles; the blue and green arrows indicate mononuclear diploid and mononuclear tetraploid cells, respectively. Graph (**c**) reports the symmetrical data distribution of mononuclear diploid, binuclear and mononuclear tetraploid hepatocytes. In the graph, the orange dot indicates the outlier, the (x) indicates the mean value and the line represents the median value for each experimental group subdivided using polyploid hepatocytes.

The haematoxylin-eosin staining showed that the cytoarchitecture of BTBR livers was well preserved, except for small signs of inflammation; the hepatocytes presented microvesicular steatosis morphology (hepatocellular ballooning). In addition, increased numbers of Kupffer cells and fat-storing cells were preserved in the parenchyma (Figure 1a). Furthermore, numerous hepatic cells exhibit crenulated nuclei that are irregularly shaped, as well as many nucleoli. Figure 1b shows the cytoarchitecture of the CTR liver for comparison.

In BTBR livers, most nuclei were diploid, either present in mononuclear diploid (MD) hepatocytes or in binuclear diploid (BD) hepatocytes (Figure 1a). Mononuclear tetraploid (MT) hepatocytes were relatively sparse (Figure 1a), whereas in CTR livers, most hepatocytes were MT (Figure 1c). Most hepatocytes in BTBR mice were MD, and only a few of them were identified as MT cells (Figure 1a). 

The CTR group showed normal liver morphology (Figure 1b). Hepatocytes display central nuclei with a regular shape and eosinophilic cytoplasm with few lipid droplets (Figure 1b). No clear histopathological changes and no inflammatory cell infiltration were observed in the liver of the CTR group. Several hepatocytes were MT cells, a few of them were BD cells, and a small number were identified as MD cells (Figure 1b).

As mentioned above, numerous BD hepatocytes were found in BTBR mice compared with MD and MT cells in the same animals. This finding was evaluated using quantitative analysis, and the results showed that BTBR mice had three times more BD cells than CTR mice (Figure 1c). After this evaluation, we also observed MD and MT cells in both experimental groups. The quantitative analysis demonstrated a significantly higher number of MD cells in BTBR mice than in CTR mice; instead, the number of MT cells appeared to be significantly lower in BTBR mice compared to CTR mice, as reported in Figure 1c.

**Figure 2:**
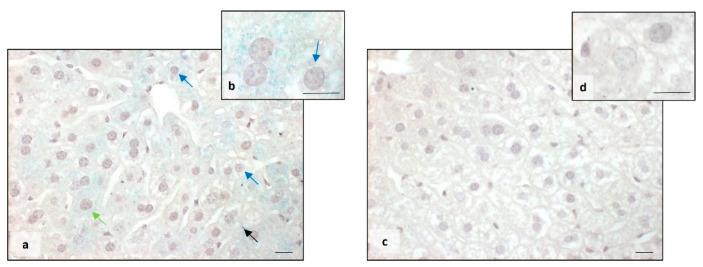
Evaluation of iron accumulation. Representative photomicrographs stained with Perls staining of BTBR (**a**,**b**) and CTR (**c**,**d**) liver. Bar: 10 µm. The black, blue and green arrows showed Kupffer cells, mononuclear diploid and mononuclear tetraploid cells, respectively, positive to Perls staining in BTBR mice.

The Perls staining showed that BTBR mice presented a higher accumulation of iron granules (blue) in the cytoplasm of MD and MT cells. Several blue granules were also observed in Kupffer cells (Figure 2a,b). On the other side, in the liver tissue of CTR mice, we observed no presence of iron either in the cytoplasm of all types of hepatocytes or in Kupffer cells (Figure 2c,d).

### 2.2. Liver Oxidative Stress, Inflammation and the Regulatory Pathways of Ferroptosis

To investigate oxidative stress and inflammation in the liver of BTBR mice, we evaluated the expression of catalase (CAT), superoxide dismutase-1 (SOD-1), GPX4 and interleukin-1 β (IL-1β).

MD, BD and MT cells showed the same expression of the abovementioned proteins in both animal groups; therefore, we identified these cells as hepatocytes.

**Figure 3:**
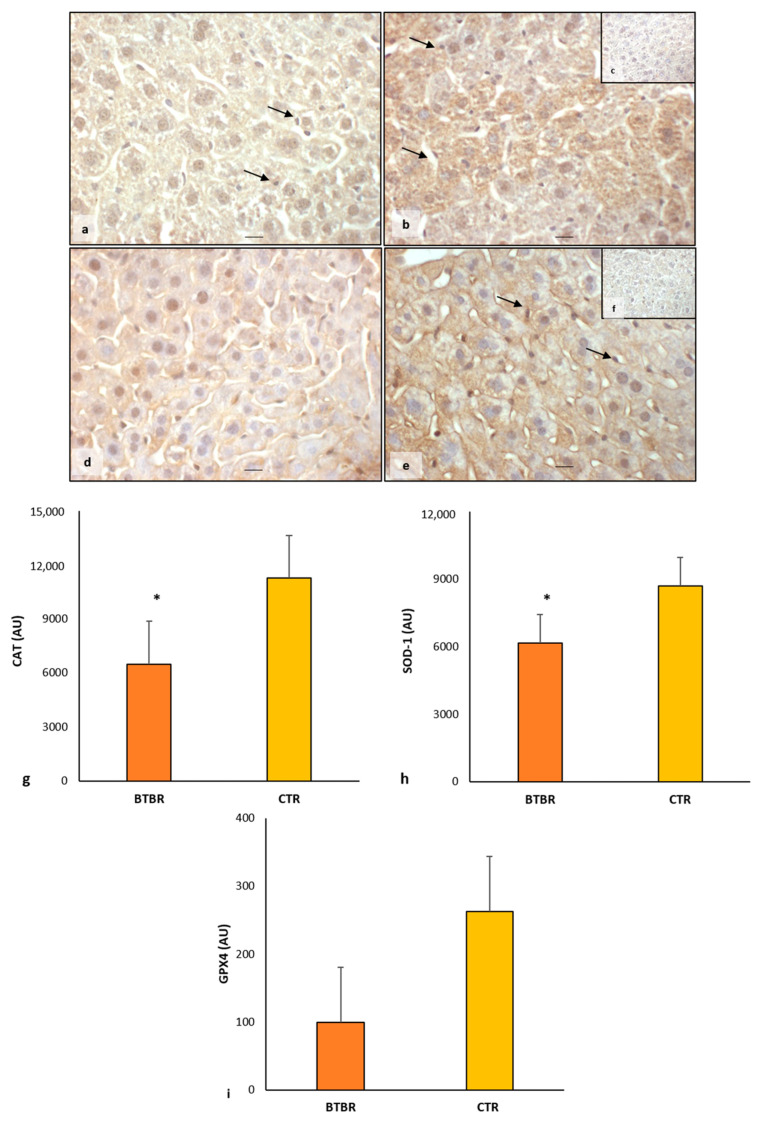
Evaluation of oxidative stress. Representative liver photomicrographs of CAT immunostaining of BTBR (**a**) and CTR (**b**,**c**) mice. Representative liver photomicrographs of SOD-1 immunostaining of BTBR (**d**) and CTR (**e**,**f**) mice. CAT (**c**) and SOD-1 (**f**) representative photomicrographs of the negative control are reported as inserts. The black arrows indicate Kupffer cells. Bars: 10 µm. The graphs summarize liver CAT (**g**), SOD-1 (**h**) and GPX4 (**i**) immunomorphometric measurement. * *p* < 0.05 vs. CTR mice. (AU): arbitrary units.

About CAT expression, in BTBR mice, we observed a weak/moderate immunopositivity in the cytoplasm of all hepatocytes (Figure 3a). Kupffer cells were negative and sometimes very weakly positive. Instead, CTR mice showed moderate/strong cytoplasmic immunopositivity in all the cells of the hepatic parenchyma, including Kupffer cells (Figure 3b).

Regarding SOD-1 expression, we observed a very weak/weak positivity in the hepatic parenchyma of BTBR mice, including Kupffer cells. This positivity was demonstrated in the cytoplasm with a scattered distribution and sometimes in the nuclei (Figure 3d). In CTR mice, SOD-1 showed a moderate/strong immunopositivity in the cytoplasm of all hepatic cells, as shown in Figure 3e. In particular, the expression of SOD-1 was very weak/negative in the nuclei of the hepatic cells of CTR mice.

The negative controls of CAT and SOD-1 immunohistochemistry were similar in both BTBR and CTR hepatic samples, and the inserts in Figure 3c,f showed a control reaction in a representative CTR liver tissue.

Comparing BTBR mice and CTR mice, we observed that in BTBR animals, both CAT and SOD-1 immunopositivity were significantly decreased compared to CTR mice. These data were confirmed through immunomorphometric analysis, as shown in Figure 3g,h.

Regarding the immunopositivity of GPX4, we observed very weak cytoplasmic positivity in BTBR mice compared with CTR mice, which showed moderate/strong positivity in liver parenchyma cells. GPX4 expression was decreased in BTBR mice compared with CTR mice, as summarized in Figure 3i.

We also evaluated the expression of IL-1β in both animal groups.

**Figure 4:**
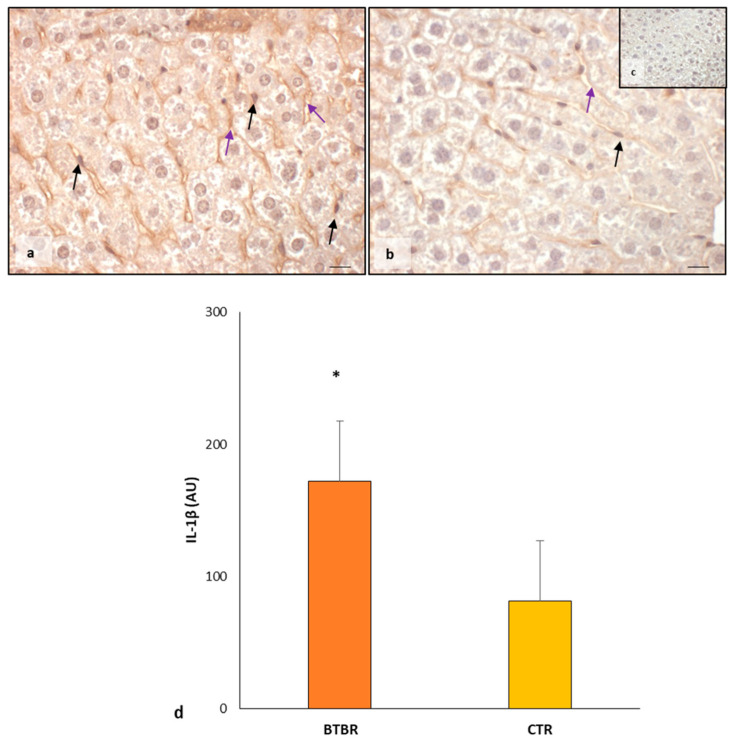
Evaluation of inflammation. Representative liver photomicrographs of IL-1β immunostaining of BTBR (**a**) and CTR (**b**,**c**) mice. IL-1β (**c**) representative photomicrograph of the negative control is reported as an insert. The black and purple arrows indicate Kupffer cells and sinusoids, respectively. Bar: 10 µm. The graph (**d**) summarizes liver IL-1β immunomorphometric measurement. * *p* < 0.05 vs. CTR mice. (AU): arbitrary units.

In BTBR mice, the weak/moderate/strong immunopositivity for IL-1β was scattered and diffuse in the cytoplasm of hepatocytes, Kupffer cells and sinusoidal cells (Figure 4a). Only rarely were the nuclei of all hepatic cells positive for IL-1β. The expression of this protein was also observed in the cytoplasm of sinusoidal cells, and it was weak or sometimes moderate (Figure 4a). The hepatocytes of CTR mice showed a very weak cytoplasmic immunopositivity for this interleukin. The nuclei of hepatocytes and Kupffer cells were negative for IL-1β. Very weak or negative positivity of IL-1β was observed in the cytoplasm of sinusoidal cells (Figure 4b).

The negative controls of IL-1β immunohistochemistry were similar in both BTBR and CTR samples, and Figure 4c shows a control reaction in a representative CTR liver tissue.

As summarized in Figure 4d, IL-1β expression was significantly upregulated in BTBR mice compared with CTR mice. 

Then, we studied KEAP1 and NRF2 as the negative regulators of the ferroptosis pathway. Furthermore, to evaluate the effects of NRF2 translocation in the nucleus as a potential trigger of cytoprotective enzyme transcription, we studied the HO-1 enzyme.

It is important to remember that MD, BD and MT cells showed the same expression of the abovementioned proteins in both animal groups; therefore, we identified these cells as hepatocytes.

**Figure 5:**
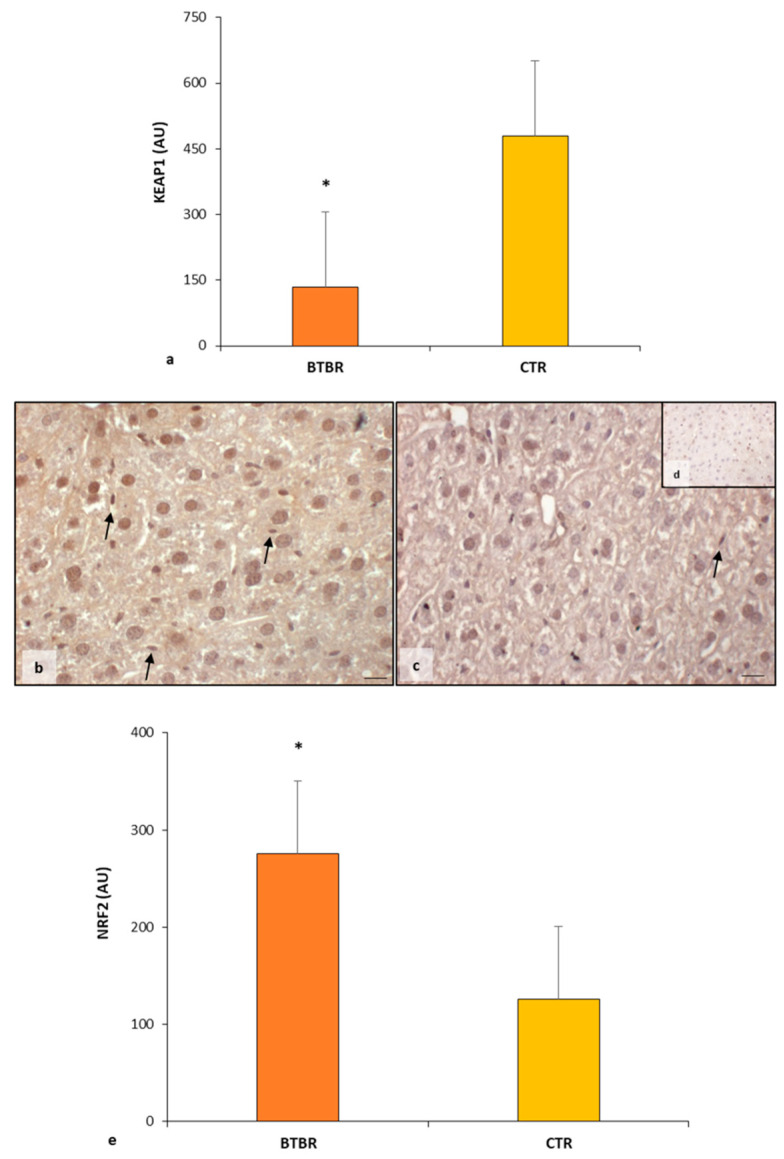
Regulatory pathways of ferroptosis. Graph (**a**) summarizes KEAP1 immunomorphometric measurement in BTBT and CTR mice. Representative photomicrographs of NRF2 immunostaining in liver sample of BTBR (**b**) and CTR (**c**,**d**) mice. NRF2 (**d**) representative photomicrographs of the negative control are reported as an insert. The black arrows indicate Kupffer cells. Bar: 10 µm. Graph (**e**) summarizes liver NRF2 immunohistochemical measurement of BTBR and CTR. * *p* < 0.05 vs. CTR mice. (AU): arbitrary units.

The KEAP1 immunopositivity in BTBR mice liver was very weak in the hepatocytes cytoplasm compared with CTR animal livers, which showed a strong/moderate expression of this protein. The immunomorphometric analysis confirmed that KEAP1 was weakly expressed in BTBR mice compared with CTR mice, as summarized in Figure 5a.

About NRF2, its cytoplasmic expression was moderate in BTBR mice, but it was very strong in almost all the nuclei of hepatocytes and Kupffer cells (Figure 5b). In fact, a few nuclei were negative for the NFR2 protein. CTR mice showed moderate/weak immunopositivity in the cytoplasm of all hepatocytes, but their nuclei were weakly positive or negative for this protein (Figure 5c). No positivity was observed in the sinusoidal cells in both groups.

The negative controls of NRF2 immunohistochemistry were similar in both BTBR and CTR samples. Figure 5d shows a control reaction in a representative CTR liver tissue. 

NRF2 expression was significantly higher in the hepatic parenchyma of BTBR mice compared with the CTR group, as reported in Figure 5e. 

The nuclear presence of NFR2 stimulates the transcription of some genes responsible for inducing the production of proteins with protective effects against the ferroptosis pathway. It is known that one of these proteins is HO-1; therefore, we evaluated this protein through immunohistochemistry and immunomorphometric analyses.

**Figure 6:**
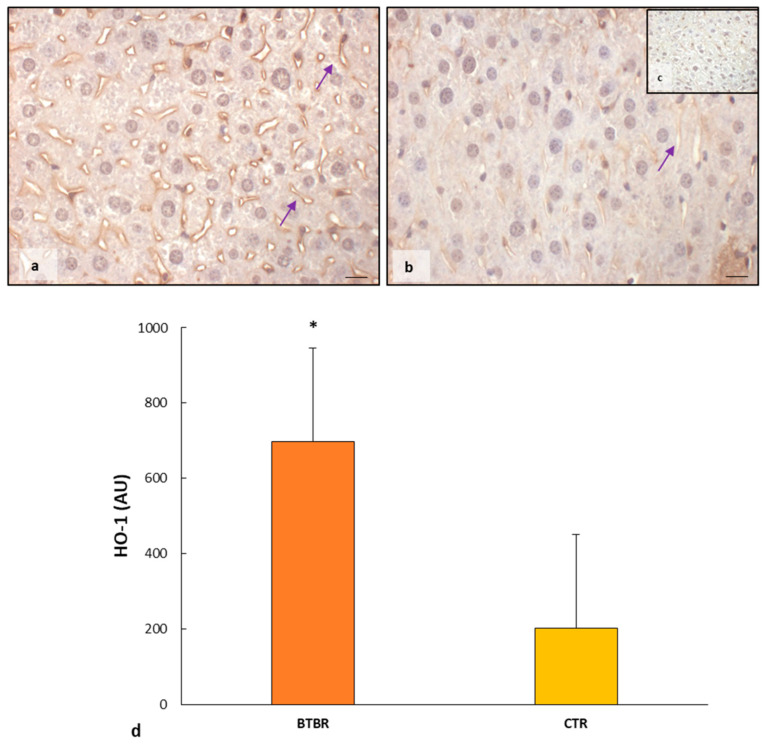
Evaluation of HO-1 in mice liver tissue. Representative photomicrographs of HO-1 immunostaining in the liver sample of BTBR (**a**) and CTR (**b**,**c**) mice. HO-1 (**c**) representative photomicrographs of negative control are reported as insert. The purple arrows indicate sinusoids. Bar: 10 µm. The graph summarizes liver HO-1 (**d**) immunomorphometric measurement comparing BTBR and CTR. * *p* < 0.05 vs. CTR mice. (AU): arbitrary units.

As observed in Figure 6a, the hepatocytes had a moderate/weak/very weak expression of HO-1 together with sinusoidal cells and Kupffer cells. CTR livers presented very weak/negative HO-1 immunopositivity in all hepatic cells, including sinusoidal cells (Figure 6b). No nuclear positivity was observed in hepatic cells in both experimental groups. 

The negative controls of HO-1 immunohistochemistry were similar in both BTBR and CTR samples, and Figure 6c shows a control reaction in a representative CTR liver tissue. 

The immunomorphometric analysis of HO-1 immunostaining was significantly upregulated in BTBR mice compared with CTR ice (Figure 6d). 

### 2.3. Melatonin Effects in the Liver of BTBR and CTR Animals

After demonstrating morphological and biochemical alterations in the hepatic parenchyma of BTBR compared with the CTR mice of the same age, we continued our study on the liver of BTBR mice treated with melatonin during the same period together with the previously mentioned groups, as reported in the Section 4 Materials and Methods. 

#### 2.3.1. Histological Evaluation of Hepatic Tissue in BTBR after Melatonin Treatment

**Figure 7:**
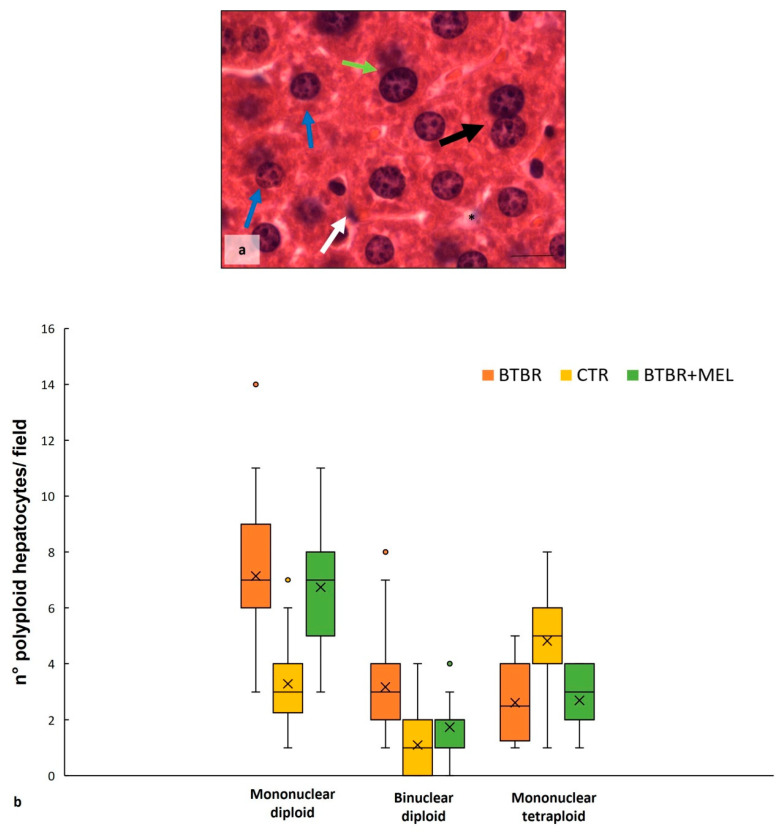
Hepatic histological evaluation. Representative photomicrographs (**a**) of the liver in BTBR mice treated with melatonin and stained with haematoxylin-eosin. Bar: 10 µm. The black and white arrows show binuclear hepatocytes and Kupffer cells respectively; the asterisks show vacuoles; the blue and green arrows indicate mononuclear diploid and mononuclear tetraploid cells, respectively. Graph (**b**) reports the symmetrical data distribution of mononuclear diploid, binuclear and mononuclear tetraploid hepatocytes. In the graph, the orange dot indicates the outlier, the (x) indicates the mean value, and the line represents the median value for each experimental group subdivided using polyploid hepatocytes.

Comparing BTBR animals treated with melatonin to BTBR mice, we observed an improvement in hepatic morphology. The liver had normal hepatocytes, some Kupffer cells and a reduction in vacuolization (Figure 7a). Regarding the number of BD, MD and MT cells, the quantitative analysis and the results demonstrated a significant decrease in BD cells, a nonsignificant decrease in MD cells, and a nonsignificant increase in MT cells compared to BTBR mice (Figure 7b).

**Figure 8:**
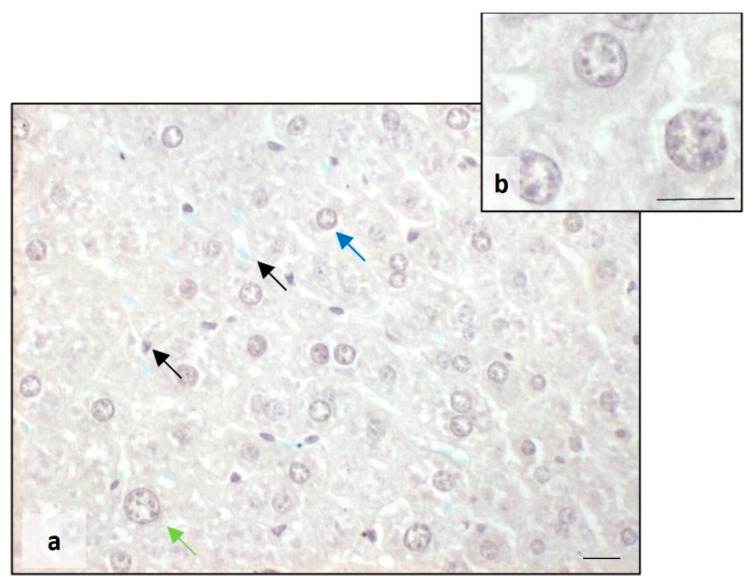
Evaluation of iron accumulation. Representative photomicrographs stained with Perls staining of the liver in BTBR mice treated with melatonin (**a**,**b**). Bar: 10 µm. The blue, green and black arrows showed mononuclear diploid, mononuclear tetraploid and Kupffer cells, respectively.

As far as the overaccumulation of iron in the cytoplasm of hepatocytes of BTBR animals, Perls staining showed a reduction in iron in the same mice strain treated with melatonin (Figure 8a,b), resulting in a normalization of the amount of iron similar to the liver tissue of the CTR group (Figure 2). We noted the presence of iron only in Kupffer cells (Figure 8a).

#### 2.3.2. Oxidative Stress, Inflammation and Regulatory Pathways of Ferroptosis in BTBR Mice after Melatonin Treatment

As previously reported, no difference in the positivity of markers was observed in the types of hepatocytes, so we considered them only as hepatocytes.

**Figure 9:**
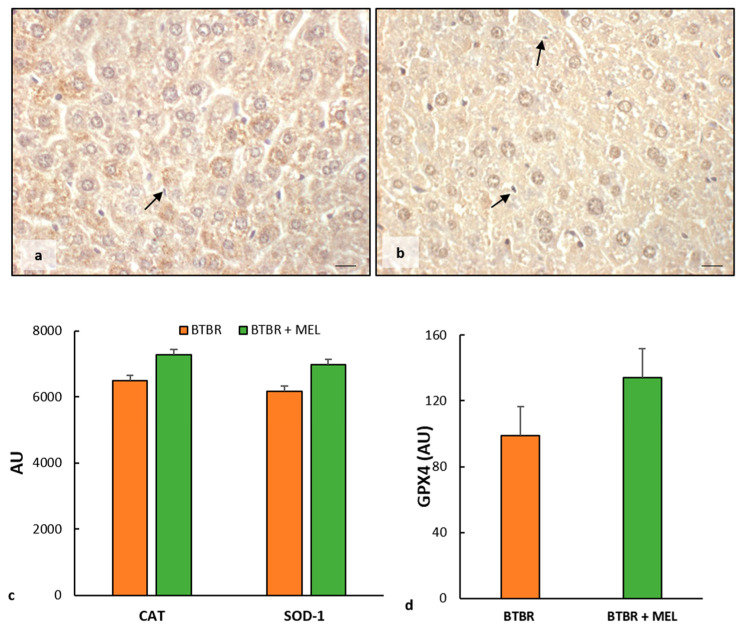
Evaluation of oxidative stress. Representative liver photomicrographs of CAT immunostaining of BTBR mice treated with melatonin (**a**). Representative liver photomicrographs of SOD-1 immunostaining in BTBR mice treated with melatonin (**b**). The black arrows indicate Kupffer cells. Bar: 10 µm. The graphs summarize liver CAT and SOD-1 (**c**) and GPX4 (**d**) immunomorphometric measurements. (AU): arbitrary units.

BTBR mice treated with melatonin showed moderate cytoplasmic immunopositivity for CAT and SOD-1 in all hepatic cells, including Kupffer cells (Figure 9a,b). The expression of these markers was likely to that observed in the CTR group and reported in Figure 3b,e.

The negative controls of CAT and SOD-1 immunohistochemistry were similar in all the samples and are reported in the inserts (c,f) of Figure 3. 

These observations were confirmed using the immunomorphometric analysis, as reported in Figure 9c, even though the increase does not show a significant trend compared to BTBR mice.

Finally, we observed a weak positivity for GPX4 in the hepatocyte cytoplasm of BTBR treated with melatonin, showing an upward trend compared to untreated mice, as reported in Figure 9d. 

We also evaluated the expression of IL-1β in BTBR mice treated with melatonin. 

**Figure 10:**
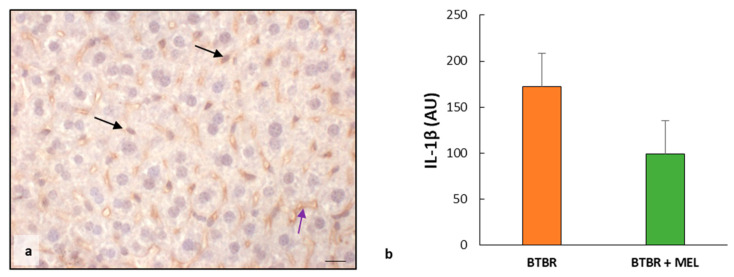
Evaluation of inflammation. Representative liver photomicrographs of IL-1β immunostaining of BTBR mice treated with melatonin (**a**). The black and purple arrows indicate Kupffer cells and sinusoids, respectively. Bar: 10 µm. The graph (**b**) summarizes IL-1β immunomorphometric measurements. (AU): arbitrary units.

We observed weak IL-1β immunopositivity in the cytoplasm of hepatocytes and sinusoidal cells; instead, the hepatocytes nuclei and Kupffer cells were negative for this interleukin (Figure 10a). The negative control of IL-1β immunohistochemistry was reported above as the insert in Figure 4c.

The immunomorphometric analysis, summarized in Figure 10b, confirmed that IL-1β immunopositivity, was significantly decreased in BTBR mice treated with melatonin compared to untreated BTBR mice. 

The immunohistochemical analysis of BTBR mice treated with melatonin showed a weak cytoplasmic positivity of KEAP1 in all hepatocytes, similar to untreated mice. 

**Figure 11:**
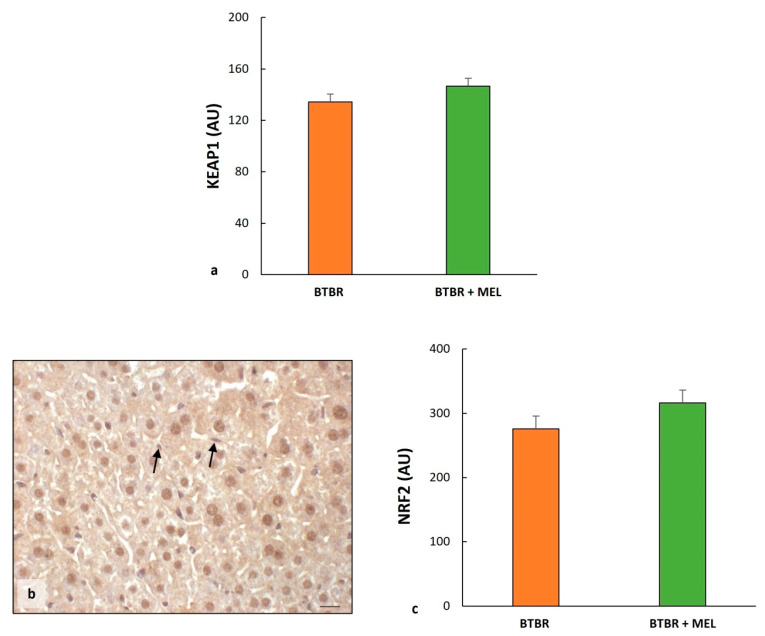
Regulatory pathways of ferroptosis. Plot (**a**) summarizes KEAP1 immunomorphometric measurements in BTBT mice and BTBR animals treated with melatonin. Representative photomicrographs of NRF2 immunostaining in the liver sample of BTBR treated with melatonin (**b**). The black arrows indicate Kupffer cells. Bar: 10 µm. Graph (**c**) summarizes liver NRF2 immunohistochemical measurements of BTBR mice and BTBR animals treated with melatonin. (AU): arbitrary units.

The immunomorphometric analysis confirmed that KEAP1 did not differ significantly between BTBR mice treated with melatonin and untreated BTBR mice (Figure 11a).

The nuclei of the cells in the hepatic parenchyma of BTBR mice treated with melatonin are moderately/strongly positive to NFR2; only some nuclei of Kupffer cells were negative (Figure 11b). No positivity was observed in the sinusoidal cells. The negative control of NRF2 immunohistochemistry was reported above as the insert in Figure 5d.

The immunomorphometric analysis showed that NRF2 tended to increase compared to untreated mice (Figure 11c) and significantly improved compared to CTR mice (Figure 5c). 

As for the evaluation between BTBR and CTR groups, we studied HO-1 protein to verify the effect of melatonin in ASD mice treated with antioxidants.

**Figure 12:**
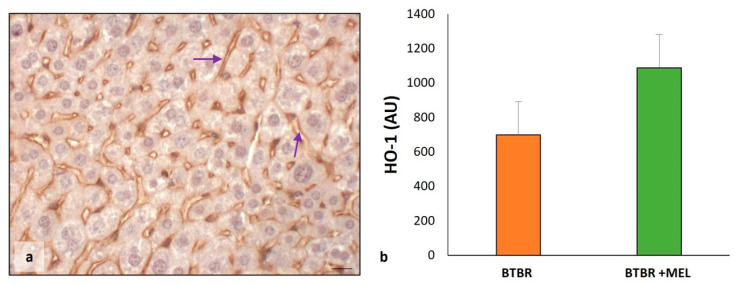
Evaluation of HO-1 in mice liver tissue. Representative photomicrographs of HO-1 immunostaining in liver sample of BTBR mice treated with melatonin (**a**). The purple arrows indicate sinusoids. Bar: 10 µm. The graph summarizes HO-1 (**b**) immunomorphometric measurements, comparing BTBR mice and BTBR animals treated with melatonin. (AU): arbitrary units.

The results showed an increase in HO-1 protein in the sinusoidal cells and cytoplasm of hepatocytes (Figure 12a) compared to untreated BTBR and to the CTR group, as reported in Figure 6. This finding was confirmed through immunomorphometrical analysis, showing a significant increase in this protein compared to untreated BTBR mice (Figure 12b). 

## 3. Discussion

Our study shows that BTBR mice at the age of 16 weeks have significantly higher numbers of MD and BD cells and fewer MT hepatocytes in their livers compared with CTR mice at the same age. Moreover, we show that oxidative stress and inflammation are involved in the liver pathogenesis of ASD in BTBR mice. A major finding in our study is that ferroptosis, a type of programmed cell death, occurs in the livers of this autistic mouse model, which is similar to defects in the neurological development of ASD [2,40]. Furthermore, we demonstrate that the antioxidant melatonin ameliorates morphological and biochemical alterations in the liver of BTBR mice.

The liver has a remarkable potential to generate new tissue in response to injury due to the proliferative ability of the parenchyma [41]. Hepatocytes are physiologically long-lived cells, similar to neurons and cardiomyocytes [42,43]. Guidotti et al. [44] demonstrated that rat liver consists almost solely of MD hepatocytes during the first postnatal 22 days; thereafter, between the age of 22 and 28 days postnatally, this MD population decreases with a concomitant increase in BD hepatocytes. Moreover, at 30 days postnatally, MT hepatocyte numbers increase, and the MD and BD populations slowly decline. Based on these findings, we propose that there are defects in liver organogenesis in BTBR mice since these mice have large amounts of MD and BD hepatocytes that were inhibited in the formation of MT hepatocytes, as was found in CTR animals at the same age. We consider this defect in the livers of these mice comparable to those observed in hippocampal neurogenesis in various animal ASD models and ASD patients [2,18,26,44,45,46,47].

Our study shows that oxidative stress and inflammation are involved in ASD, showing a downregulation of CAT, SOD-1 and GPX4 expression and an upregulation of Il-1β expression. Hepatocellular ballooning and iron accumulation in hepatocytes and sinusoidal cells were found in the livers of BTBR mice but not in CTR mice. Decreased expression of antioxidant proteins has been demonstrated in patients in relationship with ASD and neurodegenerative disorders, including Alzheimer’s and Parkinson’s diseases. In these patients, the levels of GPX4 and other antioxidant proteins, as well as the expression of their regulatory genes, are usually in dyshomeostasis [48]. Moreover, oxidative stress has been reported in connection with decreased antioxidant enzymes, increased lipid peroxidation and advanced glycation products in peripheral blood [12,49]. In association with these phenomena, we found an accumulation of lipids and iron in the hepatic parenchyma and sinusoidal cells of BTBR mice, as detected in haematoylin-eosin and Perls stained sections, respectively.

Ferroptosis is a recently discovered iron-dependent type of cell death due to ROS accumulation with free iron deposition and lipid accumulation that results in cell death [26,50,51]. The ferroptosis pathway is triggered by the iron-catalyzed accumulation of phospholipid peroxides and reduced glutathione (GSH), which is essential to protect cells in all tissues, particularly in the liver, from ferroptosis [51,52]. We show that GPX-4 expression is remarkably reduced in the livers of BTBR mice, which can induce the ferroptosis pathway. 

The transcription factor, NFR2, a master regulator of antioxidant responses and inducible cell defense systems, regulates the activity of the ferroptosis pathway and the expression of lipid peroxidation-related proteins [2,31,32,53]. NFR2 activity as a transcription factor is primarily regulated through KEAP1, which acts as a cytoplasmic anchor and promotes the degradation of NFR2 [32,35,54]. Under conditions of oxidative stress, NFR2 dissociates from KEAP1 and is then translocated into the nucleus [31,54,55]. Our results confirm these findings, showing that NFR2 expression was very strong in all nuclei of hepatocytes and sinusoidal cells, such as Kupffer cells, in BTBR mice. In contrast, CTR mice showed a lower nuclear positivity of NFR2 and a moderate/weak cytoplasmic expression. KEAP1 immunopositivity followed the expression of NFR2 in the cytoplasm of BTBR mice. 

In the nucleus, NFR2 dissociated from KEAP1 affects the transcription of specific genes that contain antioxidant response elements (ARE) and cytoprotective genes. In fact, NFR2 regulates a plethora of target genes involved in the regulation of synthesis and conjugation of GSH. HO-1 and both ferritin heavy and light chains are strictly under the control of NFR2 as well [33]. To establish that NFR2 in the liver of BTBR mice induces GSH synthesis, we studied HO-1 expression; we demonstrated that it is moderately expressed in hepatocytes of the autistic mice model and very weakly expressed in CTR mouse hepatocytes.

Inhibition of ferroptosis is a challenge focused on the mitigation of ferroptosis-related diseases. Numerous ferroptosis inhibitors have been identified in recent years, showing potential for the treatment of ferroptosis through various mechanisms [35]. Recent publications showed that the supplementation of antioxidants improves symptoms of ASD related to ferroptosis in the central nervous system [1,18].

As far as we know, only a limited number of studies have considered the role of antioxidants, such as folic acid and selenium, in the brain of BTBR mice [26], but studies on the role of melatonin on the liver parenchyma in BTBR mice have not yet been published.

Therefore, we evaluated the effects of melatonin on the liver of BTBR and CTR mice. The findings suggested improved hepatic cell polyploidy, although not all differences between BTBR and CTR mice and between treatment or no treatment with melatonin were statistically significant. Moreover, markers of oxidative stress, inflammation and ferroptosis showed a clear improvement due to melatonin treatment. We hypothesize that ferroptosis can induce cell death, blocking the evolution of BD hepatocytes into MT hepatocytes.

In conclusion, our study suggests that the liver of the autistic mouse model showed morphological and biochemical alterations, including ferroptosis, as a mechanism that is not well known in hepatic disorders, such as ASD. Moreover, we suggest that melatonin has a positive effect on symptoms and hepatic organization through ferroptosis inhibitions, which may be a potential therapeutic antioxidant for autism intervention. 

These initial findings were further investigated on the role of ferroptosis mechanisms in ASD.

## 4. Materials and Methods

### 4.1. Experimental Groups

A total of 20 male BTBR T + Itpr3tf/J (BTBR) mice (JAXTM Mice Strain; The Jackson Laboratory, Bar Harbor, ME, USA) as a transgenic animal model of ASD and 20 C57BL6/J (JAXTM Mice Strain; The Jackson Laboratory, Bar Harbor, ME, USA) mice as healthy CTR mice were housed in cages (two/three animals/cage), with food and water ad libitum, starting at post-natal day 21. The animals were kept in an animal house at a constant temperature of 20 °C with a 12 h alternating light–dark cycle to minimize circadian variations. Before the start of the experiment, mice were housed in the animal facility for 1 week. All efforts were made to minimize animal suffering and the number of animals used. All the experimental procedures were approved by the Italian Ministry of Health (n° 446/2018-PR - 20/06/2018) and followed the National Institutes of Health guide for the care and the use of laboratory animals (NIH Publications No. 8023, revised 1978). The animals were randomly subdivided into two subgroups: (a) 10 animals were treated with 10 mg/kg/day per os of melatonin, and (b) 10 animals were treated daily with the melatonin vehicle. The melatonin treatment followed the procedure reported by Borsani et al. [56]. Briefly, melatonin was given in a single daily administration per os through gavage (100 µL). On the last day of the chronic melatonin treatment, all the experimental animals, as reported in our previous study [56], were tested for behavioral tests (marble burying, self-grooming, and reciprocal social interaction tests), confirming that BTRB presented typical ASD behavioral manifestations, such as deficit in social interaction and stereotyped and repetitive behaviors. Five mice from each group were deeply anesthetized (isoflurane 5%) and transcardically perfused with saline, followed by 50 mL of 4% paraformaldehyde in phosphate buffer saline (0.1 M, pH 7.4). The liver of each experimental animal was carefully removed for the subsequent morphological and immunohistochemical evaluations [57,58].

### 4.2. Sample Processing 

After removal, the liver samples were rinsed in a physiological salt solution, dehydrated in graded ethanol, and then embedded in paraffin wax following standard procedure. Serial paraffin sections (5 µm thick) of each sample were cut with a microtome. 

### 4.3. Hepatic Morphological Evaluation

Alternate sections were deparaffinized, rehydrated and stained with haematoxylin-eosin (Bio Optica, Milan, Italy) according to the standard procedure. The sections were then observed using a light optical microscope (Olympus BX50 microscope, Hamburg, Germany). According to Guidotti et al. [44], a blind examiner identified MD, BD and MT hepatocytes using the Olympus BX50 microscope at a final magnification of 1000×. The number of all polyploid hepatocytes was evaluated in 10 random fields in each liver sample. 

### 4.4. Perls Staining: Iron Accumulation

Alternate liver sections stained with the Perls kit (Bio Optica, Milan, Italy) were used to evaluate reactive ferric iron. According to the manufacturer’s instructions, liver sections were deparaffinized, rehydrated, and then immersed for 1 h in a solution consisting of potassium ferrocyanide, acid activation buffer and distilled water. Then, the sections were washed in distilled water and stained with Mayer Emallumen for 10 min. Finally, the liver sections were dehydrated, mounted and observed with the Olympus BX50 microscope at a final magnification of 400×. The iron positivity is indicated using blue staining [59,60,61].

### 4.5. Immunohistochemical Evaluation

Alternate liver sections were deparaffinized and rehydrated, then subjected to antigen retrieval in 0.01 M sodium citrate buffer (pH 6.0) in a microwave oven for two cycles of 3 min at 600 W [62]. Then, the sections were washed in Tris-buffered saline (TBS) for 5 min and incubated in 3% hydrogen peroxide for 10 min at room temperature. To demonstrate the specificity of antibodies, we used a pre-absorption test (blocking agent): 1% bovine serum albumin in 0.05% Tween 20 for 1 h at room temperature [63]. Subsequently, liver sections were incubated for 1 h and 30 min at room temperature with the following primary antibodies: mouse monoclonal anti-SOD-1 (diluted 1:100; Santa Cruz Biotechnology, Dallas, TX, USA), rabbit polyclonal anti-CAT (diluted 1:200; Abcam, Cambridge, UK), mouse monoclonal anti-IL-1β (diluted 1:100; Santa Cruz Biotechnology, Dallas, TX, USA), mouse monoclonal anti-HO-1 (diluted 1:100; Santa Cruz Biotechnology, Dallas, TX, USA), mouse monoclonal anti-GPX4 (diluted 1:1000; Proteintech, Manchester, UK), rabbit monoclonal anti-NRF2 (diluted 1300; Proteintech, Manchester, UK) and rabbit monoclonal anti-KEAP1 (diluted 1:1000; Proteintech, Manchester, UK). Then, the samples were incubated for 1 h with specific biotinylated secondary antibodies (Vector Laboratories, Newark, CA, USA) and successively conjugated with avidin-biotin peroxidase complex (Vector Laboratories, Newark, CA, USA). The reaction products were visualized using 0.33% hydrogen peroxide and 0.05% 3,3′-diaminobenzidine tetrahydrochloride (DAB) as a chromogen (Sigma, St. Louis, MO, USA). Finally, the liver sections were counterstained with Carazzi’s Emallumen (Bio Optica, Milan, Italy), dehydrated, mounted and observed with the Olympus BX50 microscope at a final magnification of 400× [64,65]. Liver sections of BTBR mice treated with melatonin were subjected to immunohistochemical analysis for the following primary antibodies: SOD-1, CAT, GPX4, NRF2 and KEAP1. Immunohistochemical negative controls were performed by omitting the primary antibody and the presence of the isotype-matched IgG.

Five sections of each sample were analyzed by a blinded examiner, and the immunostaining for each primary antibody was evaluated as integrated optical density, expressed in arbitrary units (AU), using an image analyzer [64,65]. In detail, we performed white balancing and background subtraction for all the visual fields evaluated, and then we applied a pixel quantification algorithm to calculate the positive diaminobenzidine tetrahydrocloride-stained pixels area [66,67].

### 4.6. Statistical Analysis

Results were expressed as the mean ± standard deviation. Data distribution and statistical significance of differences among the experimental groups were analyzed using a one-way analysis of variance (ANOVA one-way test corrected Bonferroni test), with the significance set up at *p* ≤ 0.05. The data distribution was symmetrical.

## Data Availability

The data underlying this article will be shared upon reasonable request to the corresponding author.
